# Exosomes from oral tissue stem cells: biological effects and applications

**DOI:** 10.1186/s13578-020-00471-7

**Published:** 2020-09-14

**Authors:** Quan Shi, Na Huo, Xing Wang, Shuo Yang, Juncheng Wang, Tong Zhang

**Affiliations:** 1 Institute of Stomatology, First Medical Center, Chinese PLA General Hospital, Beijing, 100853 China; 2 Shanxi Medical University School and Hospital of Stomatology, Taiyuan, 030001 China; 3 Shanxi Province Key Laboratory of Oral Diseases Prevention and New Materials, Taiyuan, 030001 China

**Keywords:** Exosomes, Oral tissue stem cells, Biology function, Regenerative medicine

## Abstract

As natural nanoparticles, exosomes are a type of extracellular vesicles that are enclosed by a lipid bilayer and contain various cargos, including miRNA, mRNA, DNA and proteins. Exosomes have rapidly gained attention as a highly promising cell-free therapy. Because the cargo of exosomes changes with the changes in parent cells and status, exosomes from different types of cells may exhibit different biological effects. Considering the particularity of oral tissue stem cells, their exosomes were isolated and used to examine their related biological functions and the possibility of replacing stem cells. A variety of exosomes of oral tissue stem cells were studied, and the results revealed many special biological characteristics of these exosomes and their parent cells, especially immunomodulation, osteogenesis, odontogenesis, neuroprotection, nerve regeneration, wound healing, skin regeneration and vascularization. The oral tissue stem cell exosomes may be loaded with drugs or genes and act as tools for tumor treatment. The relevant results showed that exosomes from oral tissue stem cells were potent therapeutic tools. The present review focuses on the biological function and application of oral tissue stem cell-derived exosomes.

## Introduction

Many studies have revealed the potential of stem cells in maintaining tissue homeostasis, tissue engineering, wound healing and immunotherapy [1–3]. However, the application of stem cells also has its limitations, such as mutagenic tumorigenesis, contamination, immunorejection, and ethical limitations [4, 5]. Therefore, the identification of a method to avoid the shortcomings of stem cells and make good use of its advantages has become a hot research area. Current evidence demonstrates that many of beneficial outcomes of stem cell therapy have been localized to their paracrine effects instead of cell replacement or transplanted cell differentiation, which could activate endogenous repair pathways [6–9]. As an important component of paracrine activity, exosomes play a major role in multiple aspects of cell-to-cell interactions, and they are widely involved in tissue repair and regeneration, immune regulation, and organism development [10–12]. Exosomes are extracellular vesicles (EVs) that are secreted by living cells, enclosed by a lipid bilayer and contain various cargos, including miRNA, mRNA, DNA and proteins, which range in size from 30 to 150 nm [13–15]. Exosomes represent an important mode of intercellular communication by serving as vehicles for the transfer of its bioactive cargo to recipient cells.[16] Notably, stem cell-derived exosomes mimic the therapeutic benefits of their parent cells, which avoids the parent cell shortcomings and provides the convenience of storage and transportation without the loss of its stemness property, and provides a safer alternative approach to stem cell-based therapy [6, 17, 18]. With the enormous progress in stem cell biology in the past several years, the application of exosomes in various fields rapidly gained high interest as a highly promising cell-free therapy.

Different stem cell populations with different remarkable properties were used in different areas. Oral tissue-derived stem cells recently gained more attention because these cells are rich in source and retrieved via noninvasive procedures [19, 20]. Many kinds of stem cells were isolated from oral tissue, such as dental pulp stem cells (DPSCs), stem cells from apical papilla (SCAPs), and stem cells from human exfoliated deciduous teeth (SHEDs). Numerous studies demonstrated that stem cells from oral tissue possessed many different properties compared to other tissue-derived stem cells. Studies in the past few decades showed that these unique characteristics make oral tissue stem cells perform better in some cases [21, 22]. For example, compared with the BMSCs, the gingiva-derived mesenchymal stem cells (GMSCs) proliferate faster, do not loose MSCs characteristic at higher passages, and show stronger osteogenesis capacity;[23, 24] the osteogenic differentiation ability of alveolar bone marrow mesenchymal stem cells (ABMSCs) was higher than that of femoral BMSCs;[25] DPSCs had enhanced colony-forming ability, higher proliferative, migration ability and higher expression of angiogenesis-related genes, but lower osteogenic differentiation potential compared to the adipose-derived stem cells (ADSCs) [26]. Exosomes derived from oral tissue stem cells were also studied and showed a series of unforgettable performances [27].The present review focuses on the function and application of oral tissue stem cell-derived exosomes to help examine their therapeutic potential. More important, current isolation methods can't distinguish exosomes from extracellular vesicles, so despite some articles use “extracellular vesicles” in their experiment, if the size of the EVs are in the range of exosomes, we will also include them in this review.

## Oral tissue stem cells and exosomes

The oral cavity contains various tissues, such as teeth, jaws, gingiva, oral mucous, glands, and cartilage, and most of these tissues contain stem cells (Table 1).[19] These stem cells primarily include the tooth-related stem cells DPSCs, SHEDs, GMSCs, PDLSCs, dental follicle progenitor cells (DFPCs), stem cells from the apical papilla (SCAPs), tooth germ stem cells (TGSCs), ABMSCs, and the saliva gland-related stem cells submandibular gland epithelial stem cells (SMGepiSCs) [28]. Ease of tissue harvest, low population-doubling time, plasticity, multipotential capabilities, and immunomodulatory properties make these cells suitable candidates for various therapeutic strategies [20, 29]. In some respects, these oral tissue stem cells showed a variety of good characteristics compared with BMSCs and ADSCs as we have mentioned above, which may related to the unique tissue origin of the oral cavity and the complexity of the oral environment. Moreover, the embryonic origin of DPSCs and SHEDs is the neural crest, and these cells exhibit a special neurotropic character [27]; the gingiva is characterized by markedly reduced inflammation, rapid re-epithelialization, and fetal-like scar-less healing, which endows the GMSCs with good immunoregulation and wound-healing ability [30]. All these biological characteristics have fully explained this point of unique tissue origin and environments.

**Table 1:** The cell type and tissue origin of the oral tissue stem cells

Type of stem cells derived from oral tissue	Abbreviations	Tissue origin
Dental pulp stem cells	DPSCs	Dental pulp of permanent teeth
Stem cells from human exfoliated deciduous teeth	SHEDs	Dental pulp of exfoliated deciduous teeth
Gingival mesenchymal stem cells	GMSCs	Gingival lamina propria
Periodontal ligament stem cells	PDLSCs	Periodontal ligament
Dental follicle progenitor cells	DFPCs	Dental follicle
Stem cells from the apical papilla	SCAPs	Apical papilla tissue of the permanent teeth
Tooth germ stem cells	TGSCs	Mesenchyme of third molar tooth germ during the late bell stage of tooth development
Alveolar bone marrow mesenchymal stem cells	ABMSCs	Alveolar bone
The saliva gland-related stem cells submandibular gland epithelial stem cells	SMGepiSCs	Salivary gland tissue

However, the shortcomings of the application of the stem cells mentioned above also exist in the application of oral tissue stem cells, and the exosomes isolated from oral tissue stem cells also attracted interest. Exosomes function largely via the horizontal transfer of mRNAs, miRNAs and proteins, which function via a variety of mechanisms to alter the activity of target cells [31]. Considering the particularity of oral tissue stem cells, their exosomes were also isolated and used to examine their related functions and the possibility of replacing stem cells.

Exosomes from a variety of oral stem cells were extracted, identified and studied, and these exosomes showed many similar biological characteristics as their parent cells [32–34]. Among the research about oral tissue stem cell-derived exosomes, the majority of the published literature on oral tissue stem cell-derived exosomes recapitulates, in large part, the nature and scope that the parent cells exhibited previously in in vivo and in vitro studies, such as wound healing and immunoregulation. However, related studies also reflect a new role and prospect for exosomes. For example, exosomes are natural nanoparticles that carry drugs or genes as a new method to diagnose and treat cancer [35].

## Biological function of exosomes derived from oral tissue stem cells

Exosomes contain a diverse array of signaling molecules, mRNAs, and microRNAs and release these molecules directly into the cytoplasm of target cells to modify their biology via the activation of different signaling pathways [36, 37]. Exosomes from different cell types or different cellular states, in which the cargo is also different, may have different biological effects. Exosomes derived from oral tissue stem cells act biological functions in many aspects, and some are even impressive (Table 2).

**Table 2:** Summary of the biological function of the oral tissue stem cells derived exosomes

Biological effects	Type of exosomes	Mechanism	References
Immunomodulatory effects	DPSCs-Exos	Inhibit differentiation of CD4 + T cells into Th17 Reduce secretion of pro-inflammatory factors IL-17, TNF-α Promote polarization of CD4 + T cells into Treg cells Increase release of anti-inflammatory factors IL-10, TGF-β	[39, 40, 43]
	SHEDs-Exos	Suppress the expression of IL-6, IL-8, MMP1, MMP3, MMP9, MMP13, and ADAMTS5 SHEDs-Exo-enriched miR-100 suppresses inflammation via repression of mammalian target of rapamycin Suppressed carrageenan-induced acute inflammation in mice and suppressed the activities of cathepsin B and MMPs at the site of acute inflammation Low doses of exosomes inhibited the expression of the inflammatory cytokines IL-6 and TNF-α	
Osteogenesis effects	SCAPs-Exos	Enriched osteogenesis promoted pi-RNAs target to MAPK pathway	[41–45]
	SHEDs-Exos	High ALP activity and upregulated osteogenic gene expression, including: RUNX2, OPN and OCN Contained the mRNA and proteins of Wnt3a and BMP2 activated BMP/Smad and Wnt/β-catenin signaling pathways Inhibit the apoptosis of BMSCs, promote the RUNX2 and p-Smad5 expression Inhibited adipogenesis	
	GMSCs-Exos	Promote the expression of osteogenic markers: RUNX2, VEGFA OPN and COL1A1 Combine the exosomes with the biomaterials promoted the formation of new bone spicules and blood vessels in the rat model	
Odontogenic effects	DPSCs-Exos	Upregulation of DSP, DMP-1, ALP, and RUNX2, downregulation of latent TGF-β-binding protein 1 Promoted odontogenic differentiation via the TGFβ1/Smad signaling pathway Triggered the P38/MAPK pathway and promoted the expression of the genes required for odontogenic differentiation, including DMP1 and DPP Modulate Schwann Cells migration and odontogenic differentiation Attenuated the LPS-induced cell apoptosis of odontoblast-like cells	[33, 46, 47, 49, 50]
	SCAPs-Exos	Increased the gene expression of the dentinogenic marker DSP Promoted BMMSCs-based dentine-pulp complex regeneration	
Neuroprotection and nerve regeneration	DPSCs-Exos	Protected neurons against excitotoxicity in vitro via the activation of endogenous cell survival mechanisms Upregulate the host's endogenous growth factor expression and prevent apoptosis via activation of the cell survival PI3K-B-cell lymphoma-2 pathway	[32, 37, 52–56]
	SHEDs-Exos	Suppressed 6-OHDA-induced gait impairments and slowed the number of 6-OHDA-induced contralateral rotations Significantly inhibited the secretion of TNF-α and IL-6 Reduce neuroinflammation by shifting microglia M1/M2 polarization	
	GMSCs-Exos	Promoted Schwann cell proliferation, migration and dorsal root ganglion axon growth Increase in the expression of Notch1, c-JUN, GFAP, and SOX2 Combined with the biomaterials effectively promoted tongue taste bud regeneration and peripheral nerve regeneration	
Wound healing and skin regeneration	GMSCs-Exos	Promote the re-epithelialization, deposition and remodeling of collagen and the enhancing of angiogenesis and neuronal ingrowth in the wound area Contain higher amounts of IL-1RA	[57, 58]
Angiogenic effects	GMSCs-Exos	Contain exosomal miR-210 and promoted the expression of VEGF	[45, 59, 60]
	DPSCs-Exos	HIF-1 enhanced exosomes secretion and increased the packaging of Jagged1 The addition of Jagged1-containing exosomes cultures to endothelial cells triggered transcriptional changes in Notch target genes and induced angiogenesis	
Antitumor effects	GMSCs-Exos	PTX was incorporated into GMSCs-Exos during their biogenesis Exosomes with PTX produced a significant dose-dependent inhibition of squamous cancer cell growth	[35, 62, 64]
	DPSCs-Exos	After the transduction of DPSCs with the yCD::UPRT gene via retrovirus infection, the suicide gene yCD::UPRT mRNA was packed into the exosomes cargo The exosomes were internalized via recipient tumor cells and effectively triggered dose-dependent tumor cell death in the presence of the prodrug 5-fluorocytosine	

### Immunomodulatory effects

Oral tissue stem cell exosomes exhibit diverse immunomodulatory properties and act as regulators of innate and adaptive immunity to affect the inflammatory response. An in vitro study revealed that DPSCs-derived exosomes (DPSCs-Exos) inhibited the differentiation of CD4 + T cells into T helper 17 cells (Th17) and reduced the secretion of the pro-inflammatory factors IL-17 and TNF-α.[38] DPSCs-Exos effectively promoted the polarization of CD4 + T cells into Treg cells and increased the release of the anti-inflammatory factors IL-10 and TGF-β.[38] These capabilities of DPSCs-Exos were stronger than BMSCs-Exos, except in the inhibition of the proliferation of the CD4 + T cells, which suggests DPSCs-Exos as a new therapeutic tool for the treatment of immunological diseases. MiR-100-5p was enriched in exosomes derived from SHEDs and suppressed the temporomandibular joint (TMJ) chondrocyte expression of interleukin-6 (IL-6), IL-8, matrix metalloproteinase 1 (MMP1), MMP3, MMP9, MMP13, disintegrin and metalloproteinase with thrombospondin motifs 5 (ADAMTS5) [39]. The study revealed that miR-100-5p suppressed inflammation via repression of mammalian target of rapamycin (mTOR). miR-100-5p directly targeted the mTOR 3′ untranslated region and repressed mTOR expression [39]. The SHEDs-Exos effectively protected from inflammation-induced cartilage degradation and may be a novel potential therapeutic strategy against arthritis.

Oral tissue stem cell exosomes also showed regulatory action in acute inflammation. An in vivo study showed that exosomes derived from SHEDs effectively suppressed carrageenan-induced acute inflammation in mice and suppressed the activities of cathepsin B and MMPs at the site of acute inflammation [40]. Exosomes exerted their suppressive effects gradually and at later time points compared with prednisolone. The author speculated that the possible mechanism of the anti-inflammatory activities of exosomes were related with transport of annexin A1 into the inflamed tissues, which was identified in the exosomes [40].

### Osteogenesis effects

Bone regeneration is a wide concern in the field of regenerative medicine and in the application of stem cell exosomes. As a type of mesenchymal stem cell isolated from early stage tissues during tooth root development, the piRNA expression profiles of SCAPs-derived exosomes was different from BMSCs, including 15 piRNAs that were upregulated, and 6 piRNAs that were downregulated in SCAPs-Exos [41]. The target genes of the upregulated piRNAs in SCAPs-Exos compared to BMMSC-Exos were significantly enriched in the mitogen-activated protein kinase (MAPK) signaling pathway, which plays an important role in osteogenesis [41]. However, the specific biological role of piRNAs in osteogenesis, especially the unreported piRNAs (hsa-piR-011273 and hsa-piR-007832), must be further studied.

The SHED-Exos isolated from their parent cells effectively promoted PDLSCs osteogenic differentiation in a dose-dependent manner after 3 days of osteogenic induction and exhibited deep Alizarin red staining, high alkaline phosphatase (ALP) activity and upregulated osteogenic gene expression, including: RUNX2, OPN and OCN [42]. The activation of two pathways, BMP/Smad and Wnt/β-catenin signaling pathways, contributed to this osteogenic effect, which manifested as enhanced Smad1/5/8 phosphorylation and increased nuclear β-catenin protein expression. The cargo of the SHED-Exos contained the mRNA and proteins of Wnt3a and BMP2, which mediated the osteogenic differentiation [42]. Besides, the SHEDs-Exos can promoted BMSCs osteogenesis and inhibited adipogenesis, manifested as high expression of RUNX2 and p-Smad5, decrease expression of PPARγ.[43] This osteogenesis promotion effects has been proved in a periodontitis caused bone loss model in mouse.[43]

The osteogenesis effect of GMSCs derived exosomes was also confirmed in animal experiments model. GMSCs-Exos effectively promoted GMSCs osteogenic differentiation in combination with a 3D PLA porous scaffold, such as effectively promoted calcium deposition and the high expression of osteogenic markers: RUNX2, VEGFA, OPN and COL1A1 [44]. In a rat 5 mm cortical calvaria bone defect model, both of the GMSCs and GMSCs-Exos improved bone regeneration by promoting osteogenic properties in combination with the PLA porous scaffolds [44]. The authors further treated GMSCs-Exos with a branched polyethyleneimine (PEI) solution, namely, engineered PEI-EVs. Combined with a 3D printed PLA porous scaffold, the GMSCs-derived engineer PEI-EVs effectively promoted calcium deposition and increased RUNX2 and BMP2/4 mRNA expression [44]. GO analysis demonstrated that PEI-EVs induced upregulation of all 31 identified genes involved in the regulation of ossification and 21 genes involved in the regulation of adhesion molecules. In vivo results showed that 3D-PLA + PEI-EVs scaffolds implanted in rat cortical calvaria bone defects effectively promoted the formation of new bone spicules and blood vessels [44, 45].

### Odontogenic effects

A tooth consists of complex hard and soft tissue structures, such as enamel, dentin, pulp, periodontal ligament. Although the realization of true full tooth regeneration has not been achieved, the application of oral tissue exosomes as biomimetic tools to induce odontogenic differentiation of stem cells or treatment of dental disease has great potential.

Exosomes isolated from human DPSCs cultured in odontogenic differentiation conditions (OD-Exo) effectively triggered odontogenic differentiation of DPSCs via the upregulation of DSP, DMP-1, ALP, and RUNX2 [46]. The exosomes promoted odontogenic differentiation via the TGFβ1/Smad signaling pathway in DPSCs, which contributed to the 11 times higher exosomal MiR-27a-5p. MiR-27a-5p effectively promoted the odontogenic differentiation of DPSCs and significantly upregulated TGFβ1, TGFR1, p-Smad2/3, and Smad4 via the downregulation of latent TGF-β-binding protein 1 (LTBP1), which is an inhibitory molecule in TGFβ1 signaling [46]. OD-Exos derived from DPSCs was used in another study of dental pulp regeneration and induced the odontogenic differentiation of DPSCs and BMSCs in vitro and in vivo. [33] The OD-Exos were endocytosed by DPSCs and BMSCs in a dose-dependent and saturable manner via the caveolar endocytic mechanism and triggered the P38/MAPK pathway, which promoted the expression of the genes required for odontogenic differentiation, including DMP1 and DPP. Exosomes also increased the expression of growth factors genes (GDF 10, BMP 9 and FGF 2) and transcription factors genes (RUNX 2 and OSX) in DPSCS in 3D cultures with collagen. The OD-Exos could effectively triggered regeneration of dental pulp-like tissue and improved the vascularization of the implants in vivo in a tooth root slice model with DPSCs [33]. Moreover, the SCAPs-Exos could also promote the expression of DSP and formation of mineralized nodule in BMSCs. In a immunodeficient mice model, SCAPs-Exos effectively promoted BMSCs-based dentine-pulp complex regeneration. These applications provide a a new option for potential therapeutic method for dentine-pulp complex regeneration [47].

Oral tissue stem cell exosomes were also used in the diagnosis and treatment of dental diseases. As the main source of odontoblasts, Schwann cells (SCs) migrate to the sites of injury and differentiate into odontoblasts or dental pulp cells. Exosomes extracted from the supernatants of DPSCs and LPS-preconditioned DPSCs (LPS-Exo) regulated SC proliferation and migration, stimulated SC cells, increased the expression RUNX2, DMP1, and DSP, formed mineralized nodules, and produced dentin sialoprotein [48]. Compared with the exosomes extracted from the supernatant of DPSCs, LPS-Exos exhibited a better ability to modulate SC migration and odontogenic differentiation. During the progression of caries, some odontoblasts, located close to or immediately adjacent to the caries lesions, exhibit much severer changes upon LPS stimulation, and the distal odontoblasts showed less severe changes [49]. After induction of odontogenic/osteogenic differentiation, the SCAPs were used as a kind of odontoblast-like cells. when treated with a high concentration of LPS (20 μg/mL) the cells exhibited an accelerated release of exosomes, which attenuated the LPS-induced cell apoptosis of odontoblast-like cells treated with a low concentration of LPS (1 μg/mL) [50]. These results represent a possible mechanism that exosomes protect distal odontoblasts from LPS-induced apoptosis. Simply, the severely inflamed odontoblasts at the frontier of caries secrete exosomes that are endocytosed by other mildly injured odontoblasts, then the LPS-induced apoptosis of these mildly injured odontoblasts is suppressed.

### Neuroprotection and nerve regeneration

Many nervous system diseases, including neurodegenerative diseases and nerve damage, lack effective therapies. Various types of oral tissue stem cells and their exosomes were used in the treatment of diseases of the nervous system, especially stem cells, which originate from neural crest cells [34].

For central nervous system (CNS) diseases, exosomes attracted significant interest because these molecules pass through the blood brain barrier far more easily than their parent cells [51]. DPSCs-Exos protected neurons against excitotoxicity in vitro via the activation of endogenous cell survival mechanisms in a kainic acid-induced model of neurodegeneration [32]. The exosomes effectively upregulate the host's endogenous growth factor expression and prevent apoptosis via activation of the cell survival PI3K-B-cell lymphoma-2 (Bcl-2) pathway. The anti-apoptotic and antinecrotic potential of DPSCs-Exos were better than BMSCs-Exos [37]. The intranasal administration of SHEDs-Exos on the unilateral 6-hydroxydopamine (6-OHDA) medial forebrain bundle (MFB) rat model of Parkinson’s disease effectively suppressed 6-OHDA-induced gait impairments and slowed the number of 6-OHDA-induced contralateral rotations in the apomorphine test [52]. Improvements in motor function correlated with the normalization of tyrosine hydroxylase expression in the striatum and substantia nigra. These findings may be exploited for the development of new treatment strategies against neurodegenerative diseases.

Exosomes with or without biological materials effectively promote nerve regeneration and functional recovery. SHEDs-Exos exhibited a robust response to pro-inflammatory stimuli in a dose-dependent manner and significantly inhibited the secretion of TNF-α and IL-6 by microglia [53]. The mRNA levels of M1 polarization-associated markers were decreased significantly, and the mRNA levels of M2 polarization-associated markers increased significantly in microglia. In a rat TBI model, 500 μg/mL SHEDs-Exos injected into the rat brain improved rat motor functional recovery and reduced neuroinflammation after traumatic brain injury (TBI) by shifting microglia M1/M2 polarization [53]. Schwann cells play a critical role in the regenerative potential of the peripheral nervous system (PNS) due to their rapid response to axonal injury, which evokes the reprograming of Schwann cells toward the repair phenotype. GMSCs-Exos significantly promoted Schwann cell proliferation, migration and dorsal root ganglion axon growth, which also showed a dose-dependent increase in the expression of Notch1, c-JUN, GFAP, and SOX2, which is an important negative regulator of Schwann cell differentiation/myelination and driver of Schwann cell dedifferentiation [54, 55]. In vivo experimental results confirmed that GMSCs-Exos in combination with a biodegradable chitin conduit or GelFoam exerted comparable beneficial effects on nerve regeneration and the functional recovery of injured sciatic nerves [54, 55]. The combinatory transplantation of small intestinal submucosa–extracellular matrix (SIS-ECM) applied in critical-sized tongue defect rats promoted tongue taste bud regeneration and peripheral nerve regeneration within tongue defect areas, which is an essential component for the functional innervation of regenerated taste buds [56]. However, the efficacy of GMSCs-Exos was relatively lower on taste bud regeneration than GMSCs, which may be related to the short half-life of exosomes [56].

### Wound healing and skin regeneration

Exosomes extracted from oral tissue stem cells promoted wound healing and skin regeneration, including diabetic refractory wounds. In vivo studies showed that the combination of GMSCs-Exos and chitosan/silk hydrogel effectively promoted the healing of rat diabetic skin defects via the promotion of re-epithelialization, deposition and remodeling of collagen and the enhancing of angiogenesis and neuronal ingrowth in the wound area [57]. Considering the fast and scar-less healing of the gingiva, these impressive effects may be attributed to the excellent characteristics of GMSCs and exosomes [30]. Interleukin-1 receptor antagonist (IL-1RA) is a natural inhibitor of the proinflammatory cytokine IL-1, and it modulates a variety of IL-1–related immune and inflammatory responses and contributes to the rapid wound healing of the gingiva. Compared with the skin MSCs, GMSCs produce and secrete higher amounts of IL-1RA–expressing exosomes than skin MSCs. In vitro and in vivo research revealed that GMSCs used the Fas/Fas-associated phosphatase–1 (Fap-1)/caveolin-1 (Cav-1) complex to activate SNARE-mediated membrane fusion to secrete higher amounts of IL-1RA–expressing EVs and accelerate wound healing in the gingiva and skin [58]. These results suggest that GMSCs-Exos that contain higher IL-1RA may serve as a therapeutic agent for wound healing.

### Angiogenic effects

Angiogenesis is accompanied by a variety of physiological and pathological processes, especially tissue repair and regeneration, which play an important role as transporters of growth factors, oxygen and other factors into the tissue-repair microenvironment. In an animal model of bone regeneration and skin regeneration, the effect of oral stem cell exosomes on angiogenesis was observed. The cargo of the oral stem cell exosomes may contain a variety of components that promote angiogenesis. The GMSCs-Exos promoted the expression of VEGF, which may be attributed to the exosomal miR-210, which is a microRNA that plays a critical role in cell survival and angiogenesis [45, 59]. Exosomes extracted from HIF-1 overexpression DPSCs have an increased angiogenic capacity. HIF-1 enhanced exosomes secretion and increased the packaging of Jagged1 [60]. The addition of Jagged1-containing exosomes cultures to endothelial cells triggered transcriptional changes in Notch target genes and induced angiogenesis in vitro and in vivo. [60] This angiogenesis effect is likely due to an exosomes-Notch-endothelial cell crosstalk mediated by Jagged/Notch signal regulation that does not require classical cell–cell contact, which may have applications for the treatment of ischemia-related disease [60].

### Antitumor effects

Exosomes are a natural potent drug delivery system with many desirable features: good tolerance by host organisms, very low or no toxicity, intrinsic capability to target specific tissues or cells, and low immunogenicity [16, 61]. Nano-size exosomes may be designed and modified to load antitumor components to exert antitumor effects. Considering that minimally invasive procedures are easy to expand and with high anatomical homology to treat oral neoplasia, some oral tissue stem cells and their exosomes were used as tools to treat tumors [62, 63]. Using the ability of MSCs to incorporate molecules and release into exosomes may achieve the purpose of drug delivery. For example, at a specific concentration range, GMSCs took up paclitaxel (PTX), and PTX was incorporated into GMSCs-Exos during their biogenesis [62]. Exosomes with PTX produced a significant dose-dependent inhibition of squamous cancer cell growth [62]. Gene therapy may also be used in exosome-based antitumor treatment. After the transduction of DPSCs with the yCD::UPRT gene via retrovirus infection, the suicide gene yCD::UPRT mRNA was packed into the exosomes cargo. The exosomes were internalized via recipient tumor cells and effectively triggered dose-dependent tumor cell death in the presence of the prodrug 5-fluorocytosine (5-FC) via endocytosed exosomes and intracellular conversion of the prodrug 5-FC to 5-fluorouracil [35, 64]. These results indicate that oral tissue stem cell-derived exosomes may be one approach for targeted cancer drug delivery.

## Application method of oral tissues stem cells derived exosomes

The previous summary and review demonstrated that the exosomes of oral tissue stem cells were used in various fields and played an unexpected role. However, the rational and effective application of exosomes is the basis to achieve ideal results. The use of the right exosomes in the right manner at right times produces the right result. Here, we will briefly summarize the current application methods of oral tissue stem cells derived exosomes.

### In vitro study

In cell research, exosomes are generally used as intervention factors to directly treat target cells and observe their potential biological effects. Different studies showed that exosomes of the same cell origin may work at different doses. Related literature also suggests that exosomes function in a dose-concentration-dependent manner within a certain concentration range. The cargo of exosomes contains ubiquitous and cell type-specific biological molecules, such as protein, mRNA, and microRNA. Therefore, the cargo of exosomes may change with changes in the parents cells and their status. For example, exosomes extracted from the osteogenic-induced SHEDs carried higher amounts of Wnt3a and BMP2 compared with the exosomes directly extracted from the SHEDs, and these exosomes effectively induced PDLSCs osteogenic differentiation [42]. Stimulation of DPSCs by LPS, the DPSCs-Exos had a better ability to modulate Schwann cells migration and odontogenic differentiation [50]. To give exosomes new functions, such as antitumor effects, exosomes or the donor cells may be modified to change their cargo [65]. Therefore, it is important to consider the source and state of exosomes donor cells before the choice is made for therapeutic applications.

### In vivo study

The application methods of exosomes are diverse in in vivo studies. Based on the current related research, the following methods are the main application methods for exosomes:

1. The exosomes are directly dissolved in PBS or normal saline and injected into or around the target site. The advantage is that exosomes quickly reach the injured site to perform their functions and prevent clearance by the lung, liver and other tissues and organs. This simple method also prevents possible unexpected effects of exosomes absorption by tissues from other nontraumatic sites. However, the disadvantage is that it may not be suitable for deep lesions, such as the central nervous system and organs inside the chest and abdominal cavity. It may also increase the risk of tissue injury or infection due to injection operations.
2. Direct intravenous injection after dissolution. The advantages of this approach are that exosomes may play a role in deep injured sites with blood flow. As a natural nanomaterial, exosomes exhibit weak nonspecific interactions with circulating proteins, which indicates that serum proteins would not adsorb onto the surface of exosomes after their entry into the blood stream [65]. However, the pharmacokinetics of systemically applied exosomes may be more similar to their parent cells, and namely, most of the exosomes may be removed from the circulation and body [10, 66, 67]. Therefore, the disadvantages of this method are obvious: exosomes are under the risk of removal by the body; and higher requirement of exosomes sample preparation for clinical application.
3. Combination with biomaterials. The combination of exosomes and biomaterials also attracted great interest from researchers, especially in the tissue engineering field. Many materials were used, such as hydrogel, porous PLA scaffold, Chitin Conduits, and SIS-ECM. Exosomes bind to matrix proteins, such as type I collagen and fibronectin, which enables tethering to biomaterials [33]. Some of the biomaterials allow for the control and release of exosomes and maintain its stability. Because the exosomes are static and do not reproduce in vivo [68], the controlled and slow release of exosomes helps to make full use of their functions. For bone and tooth regeneration, the biomaterials used as scaffolds may offer mechanical and three-dimensional (3D) support that favor cell adhesion, migration, and differentiation [69, 70]. For wound healing and skin regeneration, the biomaterials may keep the wound clean and moist, which accelerates wound healing [71].

## Summary and perspective

Oral tissue stem cell exosomes are of great interest and importance in the biomedical field, and the current evidence shows that they are promising as a novel cell-free therapy for various diseases and regenerative medicine, such as immune diseases, bone and tooth regeneration, neurological diseases, and wound healing. There is no doubt that the exosomes released from oral tissue stem cells perform better than exosomes from other stem cells in some aspects.

However, many mysteries about oral tissue stem cell exosomes deserve further exploration. The number of studies focused on oral tissue stem cell exosomes is relatively limited in some types of stem cells, like ABMSCs, DFPCs and TGSCs. To be used in clinical application, More in-depth research is necessary. Besides, little is known about the key molecules between exosomes from different cells and exosomes from the same cell type under different conditions. Only by understanding the specific molecular mechanism and its regulatory network, can it be effectively applied in clinical practice. Furthermore, to play an ideal role, the appropriate exosomes dosage application methods, reasonable treatment of their parent cells, and efficient exosomes purification methods require further research to achieve.

## Data Availability

The datasets used and/or analyzed during the current study are available from the corresponding author on reasonable request.

## Abbreviations

EVs: Extracellular vesicles
DPSCs: Dental pulp stem cells
SCAPs: Stem cells from apical papilla
SHEDs: Stem cells from human exfoliated deciduous teeth
DFPCs: Dental follicle progenitor cells
SCAPs: Stem cells from the apical papilla
TGSCs: Tooth germ stem cells
PDLSCs: Periodontal tissue-related stem cells periodontal ligament stem cells
GMSCs: Gingiva-derived mesenchymal stem cells
ABMSCs: Alveolar bone-associated stem cells alveolar bone marrow mesenchymal stem cells
SMGepiSCs: Saliva gland-related stem cells submandibular gland epithelial stem cells
BMSCs: Bone marrow mesenchymal stem cells
ADSCs: Adipose-derived stem cells
Th 17: T helper 17 cells
TMJ: Temporomandibular joint
IL-6: Interleukin-6
mTOR: Mammalian target of rapamycin
MAPK: Mitogen-activated protein kinase
ALP: Alkaline phosphatase
PEI: Polyethyleneimine
LTBP1: Latent TGF-β-binding protein 1
Bcl-2: B-cell lymphoma-2
6-OHDA: 6-Hydroxydopamine
MFB: Medial forebrain bundle
TBI: Traumatic brain injury
PNS: Peripheral nervous system
SIS-ECM: Intestinal submucosa–extracellular matrix
IL-1RA: Interleukin-1 receptor antagonist
Fap-1: Fas-associated phosphatase–1
Cav-1: Caveolin-1
5-FC: 5-Fluorocytosine
PTX: Paclitaxel
3D: Three-dimensional
